# Dexmedetomidine is safe and reduces the additional dose of midazolam for sedation during endoscopic retrograde cholangiopancreatography in very elderly patients

**DOI:** 10.1186/s12876-018-0897-5

**Published:** 2018-11-06

**Authors:** Osamu Inatomi, Takayuki Imai, Takehide Fujimoto, Kenichiro Takahashi, Yoshihiro Yokota, Noriaki Yamashita, Hiroshi Hasegawa, Atsushi Nishida, Shigeki Bamba, Mitsushige Sugimoto, Akira Andoh

**Affiliations:** 1 Division of Gastroenterology, Department of Medicine, Shiga University of Medical Science, Seta Tsukinowa-cho, Otsu, Shiga 520-2192 Japan; 2 Division of Clinical Nutrition, Department of Medicine, Shiga University of Medical Science, Otsu, Japan; 3 Division of Digestive Endoscopy, Shiga University of Medical Science Hospital, Otsu, Japan

**Keywords:** Midazolam, Dexmedetomidine, Cholangiopancreatography, Endoscopic

## Abstract

**Background:**

Endoscopic retrograde cholangiopancreatography (ERCP) often requires deep sedation. Dexmedetomidine, a highly selective α2-adrenoceptor agonist with sedative activity and minimal effects on respiration, has recently been widely used among patients in the intensive care unit. However, its use in endoscopic procedures in very elderly patients is unclear. In this study, we retrospectively investigated the safety and efficacy of dexmedetomidine sedation during ERCP.

**Methods:**

The study included 62 very elderly patients (aged over 80 years) who underwent ERCP from January 2014, with sedation involving dexmedetomidine (i.v. infusion at 3.0 μg/kg/h over 10 min followed by continuous infusion at 0.4 μg/kg/h) along with midazolam. For comparison, the study included 78 patients who underwent ERCP before January 2014, with midazolam alone. We considered additional administration of midazolam as needed to maintain a sedation level of 3–4, according to the Ramsay sedation scale. The outcome measures were amount of midazolam, adverse events associated with sedation, and hemodynamics.

**Results:**

The incidence of decreased SpO_2_ and median dose of additional midazolam were significantly lower in the dexmedetomidine group than in the conventional group. The minimum systolic blood pressure and minimum heart rate during and after examination was significantly lower in the dexmedetomidine group than in the conventional group. However, serious acute heart failure or arrhythmia was not noted.

**Conclusions:**

Dexmedetomidine can decrease the incidence of respiratory complications and the total dose of other sedative agents. It can be used as an alternative to conventional methods with midazolam for adequate sedation during ERCP in very elderly patients.

## Background

Sedation in gastrointestinal endoscopy helps to not only alleviate the discomfort experienced by patients but also to improve the performance of the operator [1–3]. However, sedation can cause serious complications, such as respiratory depression and heart failure. Therefore, it is necessary to consider the safety (frequency of complications) as well as the sedative effects when selecting sedatives. In particular, very elderly patients undergoing endoscopic procedures are generally prone to sedation complications [4, 5].

Currently, benzodiazepines, such as midazolam and propofol, are widely used in endoscopic treatment, and they show an increased dose-dependent frequency of respiratory depression [6–9]. Endoscopic retrograde cholangiopancreatography (ERCP) is more invasive than other endoscopic procedures, and it often requires a comparatively deep sedation [10, 11]. It is known that the combined use of sedatives having different mechanisms of action results in synergistic sedative effects [12, 13]. Thus, the dose of each drug can be decreased, and it is possible to prevent the onset of negative incidents.

Dexmedetomidine (DEX) has low analgesic and sedative effects with low respiratory depression, and its usefulness for sedation in intensive care and local anesthesia treatment has been reported in recent years [14–16]. As it is associated with low respiratory suppression, its usefulness has been reported for sedation in endoscopic treatment [17–19]; however, its effectiveness and safety are unclear in very elderly patients undergoing ERCP. In this study, we retrospectively analyzed the influence of the combined use of DEX and midazolam for sedation during ERCP in very elderly patients.

## Methods

### Patients

Between January 2014 and June 2016, 62 consecutive patients aged over 80 years received DEX for sedation during ERCP at our hospital, and they were included in the DEX group. Between April 2012 and December 2013, 78 consecutive patients received midazolam alone, and they were included in the conventional group. The patients that the baseline percutaneous arterial blood oxygen saturation (SpO_2_) was less than 90, the systolic blood pressure was 60 mmHg or less, or the heart rate was 40 beats/min or less were excluded from analysis. All patients provided informed written consent prior to undergoing ERCP. The study was conducted in agreement with the Declaration of Helsinki and received approval from the ethics committee of Shiga University of Medical Sciences and conformed to its guidelines.

### Sedation procedure

Endoscopic examination was performed by three expert gastrointestinal endoscopists. In all patients, the sedative was administered by a sedative physician (non-anesthesiologist) who was familiar with the use of sedatives. The JF-260 V system (Olympus Medical Systems, Tokyo, Japan) was used. Oxygen (2 L/min) was administered at the start of the examination. During the examination, blood pressure, heart rate, and SpO_2_ were continuously monitored, and an electrocardiogram was continuously obtained.

In the DEX group, the initial dose of DEX (Presedex, Pfizer, Tokyo, Japan) was set at 3 μg/kg/h, and after loading for 10 min, the dose was reduced to 0.4 μg/kg/h with the range described in previous reports [18, 20].Continuous infusion was carried out with the maintenance dose until the end of the examination. Additionally, 2.5 mg of midazolam (Astellas Pharma, Tokyo, Japan) was intravenously injected at the start of the examination. A single intravenous injection of 2 mg of midazolam was repeated to maintain the sedation level at 3–4, according to the Ramsay sedation scale (RSS) [21].

**Fig. 1:**
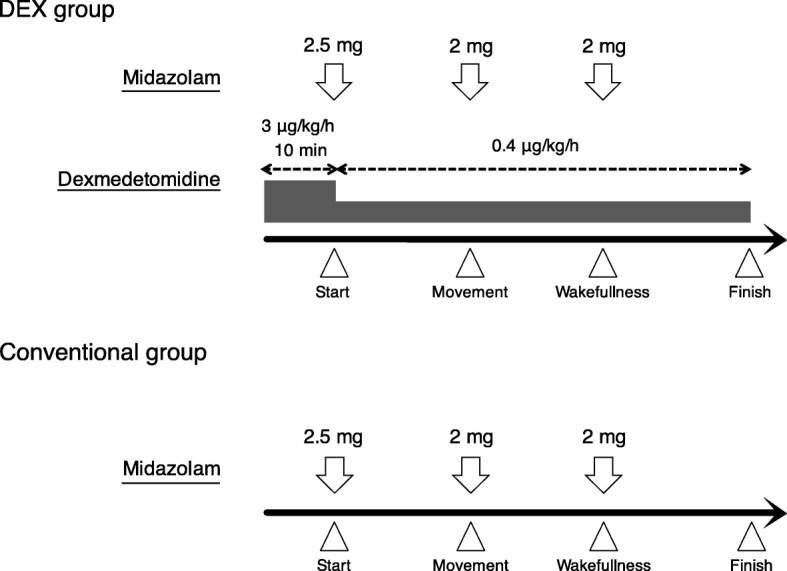
Sedative protocol in DEX group (combined dexmedetomidine and midazolam) and conventional group (midazolam alone)

In the conventional group, 2.5 mg of midazolam was intravenously injected at the start of the examination. A single intravenous injection of 2 mg of midazolam was repeated to maintain the sedation level at 4 according to the RSS, as in the DEX group (Fig. 1).

In both groups, catecholamine was administered when the systolic blood pressure was 60 mmHg or less, and atropine was administered when the heart rate was 40 beats/min or less for more than 10 s.

### Endpoints and evaluation

The main endpoint of effectiveness was the amount of midazolam. The secondary endpoint was the frequency of respiratory depression associated with sedation, the frequency of occurrence of acute heart failure and brady-arrhythmia, and time-dependent changes in blood pressure and heart rate and the frequency of administration of catecholamine and atropine. Blood pressure and heart rate were continuously measured from the start of sedation until the end of the examination, and the pre-examination values (at the time of entering the examination room), the lowest values during examination (minimum blood pressure/minimum heart rate), and the post-examination values (10 min after examination) were evaluated. Respiratory depression was defined as SpO_2_ < 90 during the examination. Procedure time was defined as the time from endoscope insertion to end of the examination.

### Statistical analysis

Measurement results are shown as median and quartile range for variables with non-normal distribution (amount of drug used) and mean ± standard deviation for variables with normal distribution (blood pressure, heart rate, and oxygen dose). Drug use was analyzed using the Mann–Whitney *U* test; background factors and complication frequency were analyzed using the chi-square test, and circulatory dynamics over time were analyzed using repeated measures analysis of variance (ANOVA). A *P*-value < 0.05 was considered to indicate a significant difference.

## Results

### Patient background

There was no significant difference between the DEX group and conventional group with regard to age, sex, body mass index (BMI), medical history, NYHA classification, reason for examination, content of enforcement treatment, and mean examination time (Table 1).

**Table 1:** Patient background

	DEX group (*n* = 62)	Conventional group (*n* = 87)	*P*-value
Age	85.2 (81–94)	85.4 (80–99)	0.30
Sex (M/F)	39/23	30/57	0.13
BMI (kg/m^2^)	21.1	19.7	0.08
Comorbidity			
Ischemic heart disease	15	23	0.85
Chronic heart failure	28	34	0.50
Arrhythmia	22	23	0.28
NYHA classification			
No cardiovascular disease	34	53	0.50
Class I	25	32	0.73
≥ Class II	3	2	0.65
Diagnosis			
Biliary stone	40	44	0.10
Biliary cancer	20	38	0.18
Others	2	5	1.0
Procedure			
EST	21	23	0.37
EPBD	12	8	0.43
ENBD	10	14	1.0
Biliary stent	24	42	0.31
Procedure time (min, mean ± SD)	45.0 ± 30.1	48.5 ± 31.2	0.94

*EST* endoscopic sphincterotomy, *EPBD* endoscopic papillary balloon dilatation, *ENBD* endoscopic nasobiliary drainage

### Comparison of total dose of midazolam

**Fig. 2:**
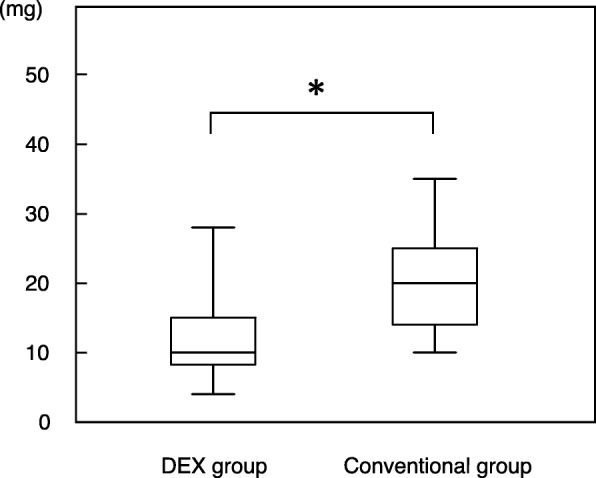
Comparison of total dose of midazolam in DEX (combined dexmedetomidine and midazolam) and conventional group (midazolam alone). *p < 0.01

The median dose of midazolam was significantly lower in the DEX group (10.0 mg) than in the conventional group (18.0 mg) (*p* < 0.001) (Fig. 2).

### The frequency of respiratory depression

The frequency of respiratory depression was significantly lower in the DEX group (0%) than in the conventional group (6.9%) (*p* = 0.04). In the two patients of the conventional group we could not continue the examination due to respiratory insufficiency.

### Sedation-related complications

Issues related to circulatory dynamics, including heart failure, sick sinus syndrome, and advanced atrioventricular block, were not observed in both groups. Atropine (0.5 mg, iv) was administered in 2 patients from the DEX group (3.2%) due to bradycardia, and in no patient from the conventional group (*p* = 0.34). Dopamine (3 μg/kg/min for 3 min) was administered because of hypotension in 1 patient from the DEX group (1.6%) and in no patient from the conventional group (*p* = 0.17) (Table 2). The three patients who had received atropine or catecholamine recovered promptly after discontinuing the administration of DEX and received all the procedure, and there was no serious respiratory or circulatory failure that would cause clinical problems during the recovery period.

**Table 2:** Sedation-related complications

	DEX group (*n* = 62)	Conventional group (*n* = 87)	*P*-value
Respiratory depression	0 (0%)	6 (6.9%)	0.04
Use atropine for bradycardia	2 (3.2%)	0 (0%)	0.34
Use vasopressor for hypotension	1 (1.6%)	0 (0%)	0.17

### Time-dependent changes in circulatory dynamics

**Fig. 3:**
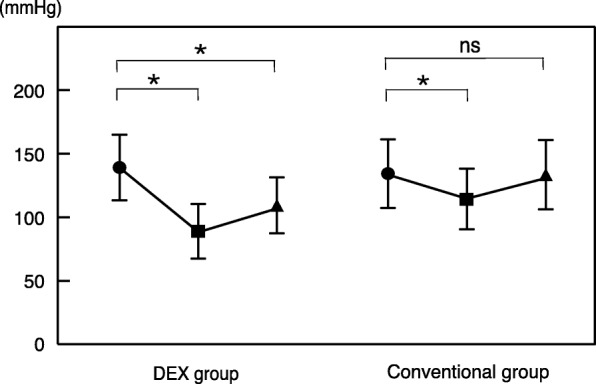
The mean lowest systolic blood pressure during the examination. The decreases were significant in both the groups when compared with the values before the examination. In the conventional group, the post-test blood pressure improved, whereas in the DEX group, the decrease in blood pressure was significantly prolonged even after the examination. * < 0.01

The mean lowest systolic blood pressure during the examination was 89.1 mmHg in the DEX group and 114.3 mmHg in the conventional group, and the decreases were significant (*p* < 0.001) in both the groups when compared with the values before the examination. In the conventional group, the post-test blood pressure improved, whereas in the DEX group, the decrease in blood pressure was significantly prolonged even after the examination (Fig. 3).

**Fig. 4:**
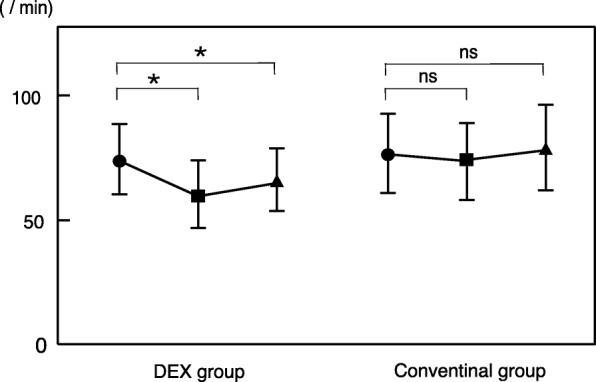
The mean lowest heart rate during the examination. In DEX group, the value was significantly lower than that before the examination. In the conventional group, the value was similar to that before the examination. In addition, in the DEX group, the decrease in heart rate was significantly prolonged even after the examination. * < 0.01

In the DEX group, the mean lowest heart rate during the examination was 62.1 beats/min, and the value was significantly lower than that before the examination (*p* < 0.001). In the conventional group, the mean heart rate was 75.4 beats/min, and the value was similar to that before the examination. In addition, in the DEX group, the decrease in heart rate was significantly prolonged even after the examination (mean 67.2 beats/min), whereas in the conventional group, the heart rate after the examination improved (mean 79.9 beats/min) (Fig. 4).

## Discussion

In this study, the combined use of DEX was found to significantly reduce the amount of midazolam, resulting in a decrease in the frequency of respiratory suppression events in very elderly patients undergoing ERCP. Although DEX tended to lower the minimum blood pressure and heart rate during examination, complications related to serious circulatory dynamics did not occur.

DEX acts on the locus ceruleus located in the pons, and it activates the α2-receptor present in noradrenaline neurons and induces sedation by suppressing upper neuron activity through negative feedback. In contrast to many other sedatives, it is characterized by limited affinity for gamma-aminobutyric acid (GABA) receptors, and therefore, there is almost no respiratory depression [21].

Although sedation by DEX has been reported for its usefulness in other endoscopic procedures, such as upper and lower endoscopy for screening purposes and endoscopic submucosal dissection (ESD), its effectiveness in ERCP is controversial. Muller et al. and Mazanikov et al. reported that it was not sufficiently effective when used alone [22, 23], while Lee et al. reported the effectiveness of the combined use of midazolam and pethidine hydrochloride in randomized trials [24]. The reason for this discrepancy is that ERCP is a more invasive procedure than other endoscopic procedures, and it is known that injection of contrast medium into the pancreatic duct and bile duct or mechanical expansion of the papilla, including endoscopic papillary balloon dilation, is painful, while mucosal resection in ESD is usually painless. DEX is considered to be somewhat weaker as a sedative than benzodiazepine and other sedative drugs [15], and a single use does not provide sufficient sedative effect for ERCP.

Conventional sedation agents, such as benzodiazepines and propofol, have been widely used for sedation in endoscopic procedures in recent years, and they have many advantages, such as good strength of the sedation effect; however, they are likely to cause side effects in a dose-dependent manner, especially the suppression of respiration [25]. Respiratory depression tends to occur in ERCP. In a prospective study using midazolam and pethidine hydrochloride, the frequency of deep sedation accompanied by respiratory depression was 86% in ERCP compared to only 26% in upper gastrointestinal endoscopy [11].

It is known that various complications tend to occur in the endoscopic treatment of very elderly patients, and it has been reported that one of the most important factors is the use of sedatives [4]. In very elderly patients, the actions of benzodiazepines and propofol tend to be excessive [5, 10, 26], and respiratory depression can be a critical complication. Although the guidelines of the American Society of Gastrointestinal Endoscopy recommend a reduction in the dose of sedative drugs in elderly patients from the viewpoint of complications [27], there is limited information on the specific use of sedatives for ERCP in very elderly patients. In this study, we demonstrated for the first time that the combined use of DEX with low respiratory depression was effective and safe for ERCP in very elderly patients.

It is known that DEX inhibits the sympathetic nerve through activation of the α2-receptor in the medulla oblongata and affects circulatory dynamics [28, 29]. In this study, the minimum systolic blood pressure and heart rate during and after the examination decreased significantly with DEX when compared to that with conventional sedation. Although catecholamine and atropine were used in some patients, we judged it was clinically acceptable as blood pressure and heart rate recovered promptly after discontinuing the administration of DEX, and there was no serious circulatory problem such as acute heart failure or severe atrioventricular block failure during the recovery period in the all patients. However, comparative study with other sedative such as propofol may be needed for more safe sedation in circulation dynamics.

In addition, DEX may not be suitable for ERCP in the case of emergency as protocol with DEX include loading time for about 10 min before the start of ERCP procedure. Improvement of the protocol with shorter loading time might solve this problem.

The present study has some limitations. This was a retrospective study performed at a single center, and it did not involve blinding. However, information bias was minimal as we used DEX in consecutive cases and adjusted the dose by setting certain criteria for reducing/adding sedatives. A prospective, randomized study is needed to clarify the appropriate sedative protocol for ERCP in very elderly patients.

## Conclusion

Dexmedetomidine can decrease the incidence of respiratory complications and the total dose of other sedative agents. Combined protocol using sedative agents with different pharmacokinetics may minimize the side effects of each. Dexmedetomidine can be used as an alternative to conventional methods with midazolam for adequate sedation during ERCP in very elderly patients.

## Abbreviations

ANOVA: Analysis of variance
DEX: Dexmedetomidine
ERCP: Endoscopic retrograde cholangiopancreatography
ESD: Endoscopic submucosal dissection
GABA: Gamma-aminobutyric acid
RSS: Ramsay sedation scale
SpO_2_: Percutaneous arterial blood oxygen saturation
